# Applying FRAME-IS to Characterize Provider-led Adaptations to a Cervical Cancer Prevention Intervention in Kenya

**DOI:** 10.21203/rs.3.rs-6933921/v1

**Published:** 2025-07-15

**Authors:** Harriet Fridah Adhiambo, Kathy Thomas, Megan M. Coe, Lynda Oluoch, Valary Ihaji, Mary Bernadette Kerubo, Alex Kinyua, Sarah Njoroge, Kenneth Ngure, Michelle Shin, Thomas A. Odeny, Bryan Weiner, Nelly Mugo, Sarah Gimbel

**Affiliations:** 1 Department of Child, Family, and Population Health Nursing, School of Nursing, University of Washington, Seattle, Washington, United States of America; 2 International Clinical Research Center, University of Washington, Seattle, Washington, United States of America.; 3 Center for Clinical Research, Kenya Medical Research Institute, Nairobi, Kenya; 4 School of Public Health, Jomo Kenyatta University of Agriculture and Technology, Nairobi, Kenya; 5 Division of Oncology, Department of Medicine, Washington University in St. Louis, St. Louis, Missouri, United States of America; 6 Department of Global Health, School of Public Health, University of Washington, Seattle, WA, USA

**Keywords:** Cervical cancer, adaptations, implementation strategies, FRAME-IS, thermal ablation, screen and treat

## Abstract

**Background:**

Implementation strategies that are contextually refined are essential for optimizing the delivery of evidence-based interventions (EBI) to prevent cervical cancer in low-resource settings. This paper reports the application of the Framework for Reporting Adaptations and Modifications to Evidence-based Implementation Strategies (FRAME-IS) to capture and disseminate strategy adaptations made to a single-visit, screen-and-treat approach with thermal ablation (SV−SAT+TA) strategy aimed at establishing sustainable cervical cancer prevention services in Kenya.

**Methods:**

A FRAME-IS-based tracking spreadsheet was developed for data collection across 10 facilities during technical assistants’ (TAs) site visits, phone calls, and monthly meetings with health providers between March 2023 and September 2024. Sources included tracking spreadsheets, TA narrative reports, and field notes from direct observations during the implementation phase. Descriptive statistics summarized site characteristics and adaptation trends. The exact Poisson test compared adaptation rates by facility level and period (early vs late).

**Results:**

A total of 28 adaptations were identified. Most adaptations (70%, n=20) occurred in the early phase. Over half were planned (57%, n=16). We made modifications to module two (What was modified). Educational adaptations were most common (57%, n=16), primarily targeting providers delivering screening and treatment services. Resources-related adaptations accounted for 21% (n=6). Additionally, 43% (n=12) of the adaptations aimed to increase adoption by expanding the number of clinicians offering the SV−SAT+TA. Nearly half (46%, n=13) targeted the organization level.

Over six months, Level five facilities had 2.67 adaptations per facility, compared to 2.85 in Level four facilities (rate ratio = 0.93 (95% CI = 0.39–2.08, p = 0.89), indicating no statistically significant difference in adaptation rates by facility levels. However, adaptation rates significantly declined, from 2.0 per facility in the early phase to 0.80 in the late phase (rate ratio = 2.50, 95% CI: 1.12–6.02, p = 0.02), suggesting a reduction in adaptations over time.

**Conclusion:**

Education and resource-related adaptations were critical to improving SV−SAT+TA implementation. Future research should focus on evaluating the impact of these adaptations on implementation and clinical outcomes, refining the FRAME-IS framework, and supporting the establishment of an adaptome to guide scalable strategies in similar settings.

**Trial registration:**

NCT05472311.

## Background

Integrating evidence-based implementation strategies in real-world settings characterized by complex contextual realities is often challenging. This complexity is heightened by misaligned organizational priorities and constrained resources, often necessitating modifications or adaptations.[1] Adaptations are deliberate alterations to an intervention or an implementation strategy aiming to achieve fit within a context while maintaining fidelity to the core components.[2,3] Evidence-based interventions and strategies are often adapted, adopted, or abandoned based on various factors, including indications of low effectiveness, emerging barriers and facilitators, and other multi-level influences.[4–6] Therefore, documenting and reporting strategy adaptations is critical to providing insights into what is more or less important for enhancing strategy effectiveness and sustainability, providing a foundation for replication in similar contexts. [1,7–9]

While we know why adaptations occur, [10,11] less is understood about their evolution, underlying patterns, and their long-term impact. These knowledge gaps limit our ability to learn from real-world implementation to optimize strategies for scale-up. This challenge is especially evident in cervical cancer prevention. Despite the availability of effective preventive interventions, the burden of cervical cancer remains on the rise in low- and middle-income countries[12] due to inconsistent implementation of these preventive measures, primarily attributed to limited resources and infrastructure.[13,14] Although most of these interventions have demonstrated success, there is limited evidence concerning context-specific adaptations made to enhance their fit and impact on clinical and implementation outcomes. This challenge is not unique to cervical cancer prevention interventions but extends to several effectiveness and implementation trials.[2,15,16]

Existing tools, such as the Framework for Reporting Adaptations and Modifications to Evidence-based Implementation Strategies (FRAME-IS), among others, provide a structured process for tracking strategy adaptations.[2,15,17] FRAME-IS has been used to track adaptations to strategies for implementing mHealth interventions,[18–20] pediatric guidelines, and mental health interventions, [7,8,18,21] yet its application remains limited in the context of cervical cancer prevention. This emphasizes the need for more empirical work to understand how strategy adaptations influence implementation.

To address these gaps, we applied FRAME-IS to capture and disseminate strategy adaptations made during the implementation of a single-visit, screen-and-treat approach with thermal ablation (SV−SAT+TA) for cervical cancer prevention in Kenya. The SV−SAT+TA is a bundled evidence-based intervention (EBI) composed of visual inspection with acetic acid (VIA) and same-day treatment with thermal ablation being implemented through a stepped-wedge cluster randomized trial in 10 health facilities in Kenya.[22–25] Henceforth, “TIBA,” a Swahili word for treatment, will represent the single-visit, screen-and-treat approach with thermal ablation. We report how FRAME-IS was used to document strategy adaptations within TIBA, providing insights necessary for its optimization and scale-up within the Kenyan health system.

## Methods

### Study setting and design

This longitudinal sub-study characterizing adaptations to implementation strategies is nested within a larger hybrid type III implementation trial aiming to develop and test implementation strategies for TIBA to enhance cervical cancer screening and treatment of precancerous lesions in Kenya (NCTO5472311).[26] TIBA was implemented across 10 health facilities in Kenya, using a three-wave stepped wedge design.

The reproductive health facilities implementing TIBA are medium to larger in size, i.e., Levels four and five [27–29] , and are located in Kiambu, Embu, and Murang’a Counties in Kenya.

### Conceptual Framework

**Figure 1:**
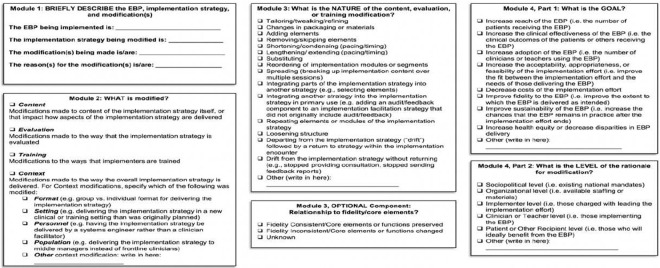
FRAME-IS Core modules

**Figure 2:**
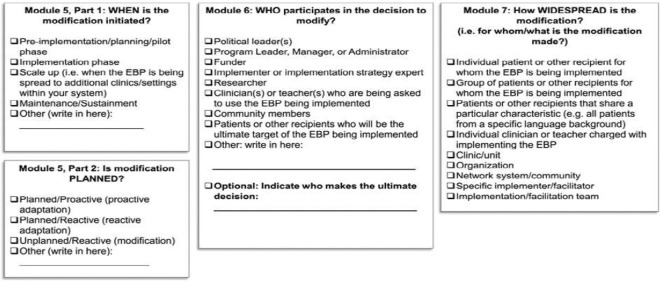
FRAME-IS Supplementary Modules

The Framework for Reporting Adaptations and Modifications to Evidence-based Implementation Strategies was employed in this sub-study to assess changes in TIBA implementation over time. FRAME-IS provides a structured, systematic approach to documenting adaptations and modifications to implementation strategies, promoting consistent reporting. [30] The framework is composed of seven modules, four of which are considered core (Figure 1). The core modules cover four main areas: a brief description of the implementation strategy (Module 1), what was modified, such as content, evaluation, content, or training (Module 2), the nature of the modification (Module 3), and the goal and level of the rationale for the modification (Module 4). Three optional/supplementary modules (Figure 2) provide additional details, including modification timing and whether the modification was planned or reactively adopted (Module 5), which participants were involved in the decision to modify (Module 6), and the extent or spread of the modification (Module 7).

We selected FRAME-IS because it is among the first and most widely employed tools developed to track adaptations to implementation strategies. Our data collection tool was informed by FRAME-IS modules 1 to 5, and we included additional sections to capture contextual factors, thus providing a comprehensive understanding of each adaptation.

### Data Collection

FRAME-IS guided the development of an Excel-based tracking tool for documenting adaptations to TIBA strategies in response to implementation barriers. These barriers to implementing TIBA and potential implementation strategies were identified during a baseline stakeholders’ workshop and through formative qualitative interviews with providers and health facility managers at each of the 10 health facilities. Each health facility was then asked to prioritize three to five key barriers to address during the implementation of TIBA, based on feasibility assessment to ensure the chosen strategies were practical and achievable within their context.

From these discussions, a list of 18 potential strategy options (Supplementary material) was generated collaboratively by the facility representatives and subsequently refined by the research team. Health facilities then applied these strategy options to address the specific barriers prioritized at each site.

**Figure 3:**
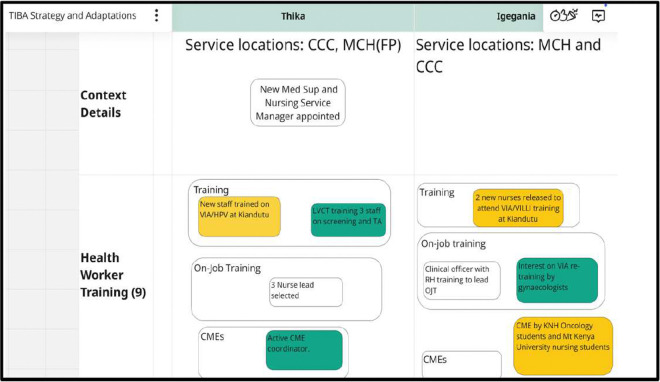
Miro Board

Data was collected monthly between March 2023 and September 2024 across the 10 health facilities, with each health facility being observed for six consecutive months during the implementation phase. The data collection occurred during technical assistants’ support visits, phone calls, and monthly meetings with health providers and their managers across the three implementation waves. The data was entered into the Excel-based tracking sheet. The data entered entailed whether the strategy component was ultimately adapted, adopted, or abandoned, a description of each adaptation, adaptation type, by whom, when it was initiated, whether it was ad hoc or planned, and the goal of the adaptation. We elicited feedback on adaptations over time through discussions with the providers and health facility managers. Additional data on contextual factors related to the strategy adaptation, barriers, and facilitators were also collected. We created a Miro board (https://miro.com) to synthesize findings and compare site adaptations. This visual whiteboard captures strategy components (clear boxes), site-specific contextual factors (green), and adaptations (yellow) that were updated monthly (Figure 3). Adaptations were considered adopted if used at two consecutive observation points.

An extraction tool aligned with the FRAME-IS modules with modifications to module 2 (What was modified-context category) of the framework to expand the categories of the adaptation types (education, resources, data management, communication, service reorganization, and community engagement) was applied by five authors (HAF, VI, BMK, AK, and SN) to systematically extract and organize the data for analysis. During the extraction phase, the five authors convened two brief meetings to assess the consistency and accuracy of their interpretations of their characterization of the adaptations. Additionally, we categorized the adaptations into two periods, i.e., early adaptations (1–3 months after the site initiated the intervention) and late adaptations (from months 4–6).

The extracted data was reviewed by an independent coder (MMC) to assess the consistency and reliability of the coding process. Three discussion meetings were held between the lead author, the independent coder, and one of the project’s investigators (SG) to review convergence, reconcile discrepancies, and reach a consensus on the characterization of the adaptations.

### Analysis

We used descriptive statistics to summarize the study sites’ characteristics and describe trends and patterns in adaptation types over time and across the health facility levels. Adaptation rates were computed per facility per 3 or 6-month period. Due to the small counts of adaptations in our analysis, we used an exact Poisson test to compare whether the adaptation rates varied across the health facility level, which we grouped as level four vs five. The test produces a rate ratio, a 95% confidence interval, and a p-value. For the same reason, we also applied the exact Poisson test to compare whether the adaptation rates significantly changed across the two time periods (early vs late). However, the exact Poisson test does not take into account the pairing in the data across time points and is, therefore, conservative. Analyses were performed using R version 4.5.0 and “*poisson. exact”* R package.

### Ethical considerations

Ethics approval was granted by the Scientific and Ethical Review Committee of the Kenya Medical Research Institute (SERU-KEMRI No. 4403) in Nairobi, Kenya, and the Human Subjects Institutional Review Board (STUDY00014200) at the University of Washington, Seattle, USA.

## Results

Overall, 70% of health facilities were Level four (n=7), and 30% were Level five (n=3). Most (80%, n=8) of the health facilities were located in urban areas and were publicly managed (70%, n=7). Two hundred and sixty health providers, including nurses, clinical officers, and gynecologists, were trained on cervical cancer screening using VIA and treatment with thermal ablation, with 58% (n=152) of the providers from Level four health facilities. Approximately 18%(n=46) of the providers trained offered screening and treatment services during the study period across both site levels. Table 1 provides a summary of the characteristics of the participating health facilities.

### Module 2: What was modified

We modified the category names of module 2 of FRAME-IS to support a more tailored categorization of the adaptation types seen in our work. A total of 28 adaptations were identified, of which two adaptation types were unique. Over half of the adaptations (57%, n=16) were related to education, targeting patients or providers, followed by resource-related adaptations (21%, n=6), for example, changes to essential commodity acquisition. Table 2 provides the detailed distribution of the adaptation types, with the education category representing the most significant proportion.

**Figure 4:**
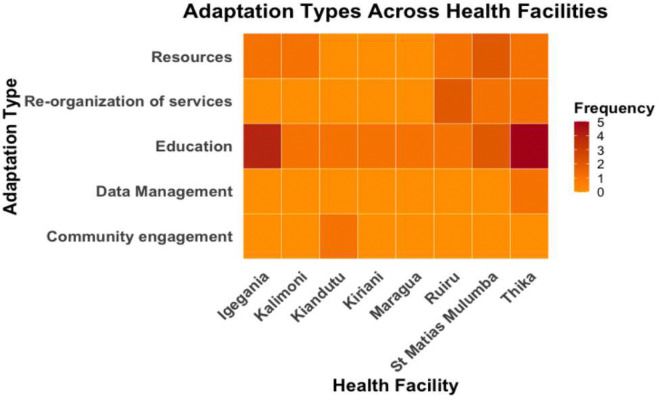
Heat map visualizing adaptation types across health facilities

Figure 4 visualizes the distribution of adaptation types across 8 health facilities that made adaptations. Overall, there is a variation in the adaptation types, suggesting that some facilities were more focused on certain adaptations than others.

### Module 3: Nature of Adaptations

Almost half of the adaptations (46%, n=13) focused on tailoring provider education related to cervical cancer. For example, new staff who missed training sessions at their own facility were allowed to attend training on cervical cancer screening at nearby health facilities to increase the adoption of the TIBA intervention at their facilities. The training strategy was tailored depending on the staffing challenges at each site. Furthermore, many educational adaptations were enhanced by adding new components (29%, n=8), including the creation of alternative patient pathways for individuals ineligible for thermal ablation and supplementary resources such as links to the International Agency for Research on Cancer (IARC) that offered in-depth information on cervical cancer to bolster provider competency.

### Module 4: Goals and levels for modifications

Of the 28 adaptations, 43% (n=12) aimed to increase the adoption of TIBA by expanding the number of clinicians offering cervical cancer screening with VIA and treatment with thermal ablation through provider training. An additional 36%(n=10) focused on increasing reach, i.e., the number of patients receiving TIBA. This was achieved by increasing the number of cervical cancer service delivery points, providing essential screening commodities, and targeting in-reach and outreach activities. Eighteen percent (n=5) targeted improving fidelity through supportive supervision, and one (3%) adaptation aimed to improve clinical effectiveness.

Most adaptations (46%, n=13) targeted the organizational level, emphasizing the leadership role in resource mobilization to ensure continuity in cervical cancer delivery. Approximately 39% (n=11) targeted the clinician level, addressing gaps in provider skills, which aligned with the goal of increasing the number of providers implementing TIBA. Adaptation at the patient level were less frequent (14%, n=4), but those focused on expanding TIBA reach.

### Module 5: Timing of the modifications and their level of planning.

**Figure 5:**
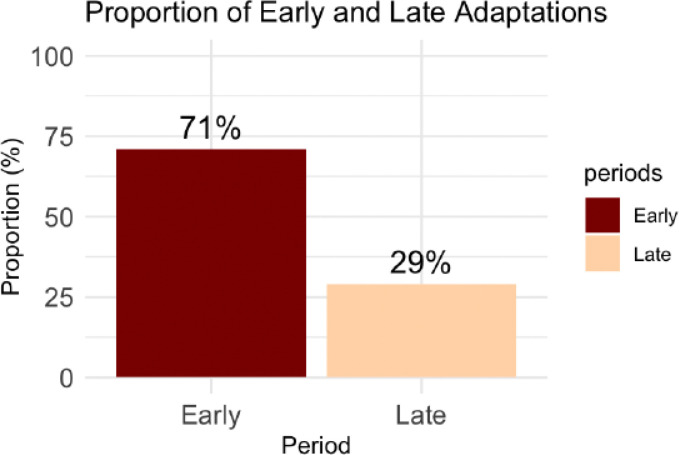
Proportion of Adaptation Timing

All the adaptations occurred during the implementation phase, and the majority occurred in the early phase (71%, n=20) (Figure 5). Over half of the adaptations (57%, n=16) were planned. A substantial proportion of unplanned adaptations were mainly implemented in response to clinical training needs among new staff and delays in the procurement of essential commodities. 75% (n=21 ) of adaptations were adopted, and none were abandoned. However, given our definition of an adopted adaptation (two consecutive observation points ), the majority (seven) of the adaptations not adopted after the first round of implementation were one-time ad-hoc changes mainly intended to address skill gaps among providers of cervical cancer screening and treatment.

### Change in adaptation rates across time and across health facility levels

**Figure 6:**
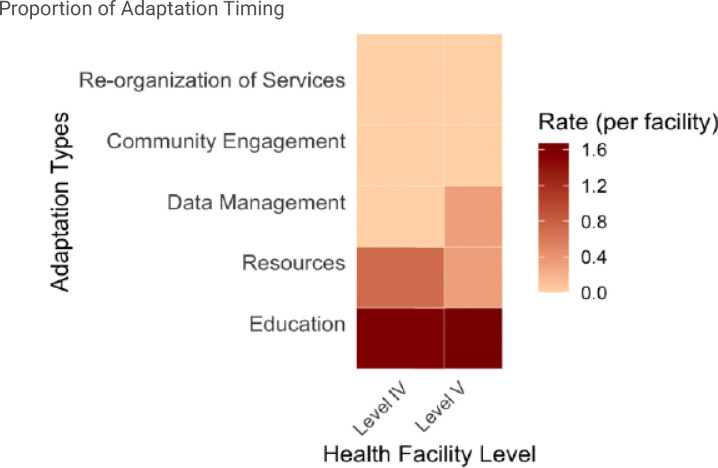
Adaptation Rates Comparing Level four and five Health Facilities.

Table 3 summarizes adaptation rates across time and Levels four and five health facilities. We observed an overall rate of 2.67 adaptations per facility over a six-month period in Level five facilities, compared to 2.85 adaptations per clinic over the same period in Level four facilities (rate ratio = 0.93 (95% CI = 0.39–2.08, p = 0.89), indicating no statistically significant difference in adaptation rates between the facility levels. Figure 6 visualizes adaptation rates across health facility levels.

During the early phase, we observed an overall rate of 2.0 adaptations per facility, compared to 0.80 adaptations per facility in the late phase (rate ratio =2.50, 95% CI = 1.12–6.02, p = 0.02), indicating a statistically significant decrease in adaptations over time.

## Discussion

Our study provides key insights into patterns of strategy adaptations necessary to enhance fit and optimize cervical cancer prevention, particularly in LMICs. Guided by FRAME-IS, we identified 28 adaptations, most of which focused on provider and patient education, followed by resource adaptations. The primary goals of these adaptations were to increase provider adoption of TIBA and reach (the number of women being screened and treated for precancerous lesions), with most of the adaptations occurring in the early phase of implementation and being planned. Adaption rates were consistent across health facility levels, though significantly higher in the early implementation phase, highlighting early enthusiasm to enhance strategy fit. Education-related adaptations aimed at tailoring strategies to staff needs and local contexts, and they remained common across the time periods (early and late periods).

We observed an overall rate of 2.0 adaptations per facility in the early phase, compared to 0.80 adaptations per facility in the late phase (rate ratio =2.50, 95% CI = 1.12–6.02, p = 0.02), indicating a statistically significant decrease in adaptations over time. Early adaptations occur as initial challenges and misalignments with context are more apparent, resulting in proactive adjustments to improve the fit of an intervention or strategy.[31] For example, in our study, challenges such as high provider workload, staff transfers between departments and clinics, and onboarding new staff are findings reported in the literature [32–34] that led to the tailoring of provider education. These adaptations, occurring early in the implementation phase, primarily focused on the action target (health providers) by offering flexible training opportunities at nearby health facilities and additional mentorship from implementing partners to ensure adoption, fidelity to the clinical task, and broader use (reach). Addressing challenges through tailored context-specific adaptations ensures better alignment with the contextual realities, enhancing strategy effectiveness and sustainability.[35,36] We hypothesize that the frequent adaptations during the early phase were necessary as TIBA fit within the health facility setting. Over time, these adaptations were successfully integrated into the health facility’s routine, marking a stabilization phase during the later phase, hence the decline in the adaptation rate over time. It is also possible that there was a decline in organizational enthusiasm.

We found no significant differences in the adaptation rates across the health facility levels. However, following Geng et al.’s [37] recommendation to consider the “dose” of an adaptation, tracking both the frequency and intensity of specific adaptations, such as provider education, could offer deeper insights into site-level variation that may otherwise go unrecognized.

Most of our adaptations were planned as recommended in the literature[38] and targeted the organizational level. This allowed the health facilities to maximize the fit of the strategies through collaborative meetings and goal setting among key stakeholders, including frontline staff/implementers, health facility managers, and implementing partners, ensuring aligned priorities and resources. The approach minimized disruptions in service delivery, emphasizing the importance of meaningful stakeholder engagement in ensuring the success of an intervention.[8,39,40]

Interestingly, two health facilities did not report any adaptations to the implementation strategies. Both were Level five health facilities located in urban areas and did not face challenges with essential commodities for cervical cancer screening, as they received support from larger implementing partners and procured these commodities through their health facilities, unlike the other sites. One of the facilities also provided training on cervical cancer screening and treatment to health providers in peripheral health facilities. This initiative aimed to reduce the number of women referred to their facility for screening and precancer treatment. It sought to increase the broader adoption of TIBA among providers and expand its reach within the region. Additionally, leadership support in these two facilities was evident, and a positive and receptive attitude was observed among providers offering cervical cancer preventive services. Perhaps these two health facilities, in addition to their leadership support, possessed a high level of adaptive capacity[41] and had a functioning system in place, possibly explaining the lack of strategy adaptations. While leaders from all the facilities were involved in the strategy development phase, contributions from these active leaders may have led to the strategy having better git when introduced, so requiring less adaptation. Further exploration is needed to examine how the clinical and implementation outcomes differed between these facilities and others with strategy adaptations.

### Lessons learned when applying FRAME-IS

FRAME-IS was valuable in documenting and tracking adaptations to the TIBA strategy during the implementation phase. It provided a clear view of how TIBA was optimized within the various health facilities.

Determining whether an adaptation occurred was challenging when the original strategy implementation plan lacked sufficient detail. Proctor’s recommendation for naming, defining, and operationalizing implementation strategies would help ensure consistency in documenting strategy adoptions and adaptations.[42]

FRAME-IS does not provide guidance on adopted strategy adaptations. We defined “adopted adaptations” as those changes that were consistently implemented over two months. We also incorporated a section in the data collection tool to capture emerging barriers and facilitators, providing actionable guidance for strategy adjustments.

FRAME-IS characterizes adaptations into four main categories, i.e., context, content, training, and evaluation, as outlined in module 2. However, during preliminary coding, we encountered challenges applying these categories, as they were not mutually exclusive, and the terminology used was unfamiliar to Technical Assistants and would subsequently be challenging to health providers during dissemination. To address this, we expanded the context and training adaptation types into six categories: community engagement, data management, resources, education, communication, and reorganization of services, which were terminologies familiar and easily interpreted among the health providers in our context. Despite these revisions, we still faced challenges coding certain adaptations. For example, patient education can logically fit under the “community engagement” and “education” categories. Similar challenges have been reported by other authors[8,18], who addressed these challenges by incorporating additional modules to provide in-depth information on the specific adaptations, implementing a numbering system to map changes to other modules, and adding supplementary coding categories. These refinements can be considered by the authors of FRAME-IS if there is a future update to the framework.

This study had limitations. First, we have not assessed the impact of these adaptations on the implementation and clinical outcomes, which would have provided insights into which adaptations were effective in optimizing TIBA. However, this does not undermine the validity of our findings, as we provided useful information on the adaptation types and examined the trends and patterns of these adaptations during TIBA implementation. Secondly, the study’s technical assistants obtained information on the adaptations through monthly conversations with providers and their managers, which may have potentially introduced recall and social desirability biases. Finally, due to time constraints, we could only track adaptations during the active implementation phase, not the maintenance phase. Perhaps tracking adaptations beyond the implementation phase would have provided additional insights into the sustainability patterns of the adaptations.

### Strengths

FRAME-IS has previously been applied in Kenya to track adaptations to a mobile health strategy[18]; however, our study is the first to report its application in the context of cervical cancer preventive intervention. We prospectively collected data on these adaptations, offering valuable learning opportunities on optimizing the framework’s use while making real-time adjustments to the data collection tool to enhance data quality. We compared adaptation rates across health facility levels and examined how these adaptations evolved. This comparison provided useful information on immediate adaptations needed to support the successful implementation of TIBA.

## Conclusion

We demonstrate the capability of FRAME-IS in documenting and tracking strategy adaptations, highlighting the critical role of education adaptations as the most frequently needed modifications to optimize cervical cancer preventive interventions. Over time, we observe a reduction in these adaptation rates, indicating a potential stabilization or strategy fit. Based on our experiences applying the framework, we offer recommendations targeting refinements to module 2 categories, adding new modules to link strategy adaptations to outcomes, and improving data capture of contextual factors influencing the implementation of the adapted strategies.

Future studies should focus on evaluating the impact of these adaptations on both implementation and clinical outcomes, exploring the “dose” of adaptations, and determining an ideal duration for tracking them. Furthermore, establishing an “adaptome,” a knowledge base, or a collection of implementation strategy adaptations[8,42] would allow the collection of contextualized data that can be easily adapted, facilitating scaling and replication of strategy adaptations in different settings.

## Supplementary Material

This is a list of supplementary file associated with this preprint. Click to download.

Table1ParticipantCharacteristics.docx

Table2AdaptationTypes.docx

Table3ChangeinAdaptationRates.docx

STROBEchecklistv4combined.docx

## Data Availability

Data is available at https://figshare.com/s/6d5220858949a872aec8

## List of abbreviations

SV−SAT+TA: Single visit, screen-and-treat approach with thermal ablation
FRAME-IS: Framework for reporting modifications and adaptations to evidence-based implementation strategies
TA: Thermal ablation
VIA: Visual inspection with acetic acid
EBI: Evidence-based interventions

## References

[1] Nguyen MX, Bartels SM, Akiba CF, Sripaipan T, Nong HT, Dang LT, Tracking modifications to implementation strategies: a case study from SNaP - a hybrid type III randomized controlled trial to scale up integrated systems navigation and psychosocial counseling for PWID with HIV in Vietnam. BMC Medical Research Methodology. 2024 Oct 26;24(1):249. 39462341 10.1186/s12874-024-02367-3 PMC11520046

[2] Haley AD, Powell BJ, Walsh-Bailey C, Krancari M, Gruß I, Shea CM, Strengthening methods for tracking adaptations and modifications to implementation strategies. BMC Medical Research Methodology. 2021 Jun 26;21(1):133. 34174834 10.1186/s12874-021-01326-6 PMC8235850

[3] Wiltsey Stirman S, Baumann AA, Miller CJ. The FRAME: an expanded framework for reporting adaptations and modifications to evidence-based interventions. Implementation Science. 2019 Jun 6;14(1):58. 31171014 10.1186/s13012-019-0898-y PMC6554895

[4] Smith JD, Merle JL, Webster KA, Cahue S, Penedo FJ, Garcia SF. Tracking dynamic changes in implementation strategies over time within a hybrid type 2 trial of an electronic patient-reported oncology symptom and needs monitoring program. Front Health Serv [Internet]. 2022 Nov 1 [cited 2025 Feb 6];2. Available from: https://www.frontiersin.org/journals/health-services/articles/10.3389/frhs.2022.983217/full 10.3389/frhs.2022.983217 PMC10012686 36925901

[5] Moore G, Campbell M, Copeland L, Craig P, Movsisyan A, Hoddinott P, Adapting interventions to new contexts—the ADAPT guidance. BMJ. 2021 Aug 3;374:n1679. 34344699 10.1136/bmj.n1679 PMC8329746

[6] Pettersson K, Liedgren P, Giannotta F, von Thiele Schwarz U. Eleven Reasons for Adaptation of Swedish Parenting Programs. Front Health Serv. 2022 Jul 11;2:923504. 36925861 10.3389/frhs.2022.923504 PMC10012651

[7] Miller CJ, Sullivan JL, Connolly SL, Richardson EJ, Stolzmann KL, Brown M, Adaptation for sustainability in an implementation trial of team-based collaborative care. Implementation Research and Practice. 2024 Jan 23;5:26334895231226197. 38322803 10.1177/26334895231226197 PMC10807389

[8] Schoenthaler A, De La Calle F, De Leon E, Garcia M, Colella D, Nay J, Application of the FRAME-IS to a multifaceted implementation strategy. BMC Health Serv Res. 2024 Jun 1;24:695. 38822342 10.1186/s12913-024-11139-0 PMC11143702

[9] Ovretveit J, Dolan-Branton L, Marx M, Reid A, Reed J, Agins B. Adapting improvements to context: when, why and how? Int J Qual Health Care. 2018 Apr;30(Suppl 1):20–3. 29878138 10.1093/intqhc/mzy013 PMC5909662

[10] Escoffery C, Lebow-Skelley E, Haardoerfer R, Boing E, Udelson H, Wood R, A systematic review of adaptations of evidence-based public health interventions globally. Implementation Science. 2018 Sep 26;13(1):125. 30257683 10.1186/s13012-018-0815-9 PMC6158804

[11] Salloum RG, Wagner TH, Midboe AM, Daniels SI, Quanbeck A, Chambers DA. The economics of adaptations to evidence-based practices. Implement Sci Commun. 2022 Sep 24;3:100. 36153575 10.1186/s43058-022-00345-8 PMC9509646

[12] Cervical cancer [Internet]. [cited 2025 Feb 12]. Available from: https://www.who.int/news-room/fact-sheets/detail/cervical-cancer

[13] Reza S, Anjum R, Khandoker RZ, Khan SR, Islam MdR, Dewan SMR. Public health concern-driven insights and response of low- and middle-income nations to the World health Organization call for cervical cancer risk eradication. Gynecologic Oncology Reports. 2024 Aug 1;54:101460. 39114805 10.1016/j.gore.2024.101460 PMC11305207

[14] Gossa W, Fetters MD. How Should Cervical Cancer Prevention Be Improved in LMICs? AMA Journal of Ethics. 2020 Feb 1;22(2):126–34. 10.1001/amajethics.2020.126 32048583

[15] Miller CJ, Barnett ML, Baumann AA, Gutner CA, Wiltsey-Stirman S. The FRAME-IS: a framework for documenting modifications to implementation strategies in healthcare. Implement Sci. 2021 Apr 7;16:36. 33827716 10.1186/s13012-021-01105-3 PMC8024675

[16] Sjoberg H, Kenney RR, Morgan B, Connelly B, Jones CD, Ali HN, Adaptations to relational facilitation for two national care coordination programs during COVID-19. Front Health Serv. 2022 Jul 22;2:952272. 36925807 10.3389/frhs.2022.952272 PMC10012763

[17] Smith JD, Norton WE, Mitchell SA, Cronin C, Hassett MJ, Ridgeway JL, The Longitudinal Implementation Strategy Tracking System (LISTS): feasibility, usability, and pilot testing of a novel method. Implementation Science Communications. 2023 Nov 28;4(1):153. 38017582 10.1186/s43058-023-00529-w PMC10683230

[18] Mangale DI, Onyango A, Mugo C, Mburu C, Chhun N, Wamalwa D, Characterizing provider-led adaptations to mobile phone delivery of the Adolescent Transition Package (ATP) in Kenya using the Framework for Reporting Adaptations and Modifications to Evidence-based Implementation Strategies (FRAME-IS): a mixed methods approach. Implementation Science Communications. 2023 Aug 14;4(1):95. 37580836 10.1186/s43058-023-00446-y PMC10424422

[19] Quimby KR, Murphy MM, Harewood H, Howitt C, Hambleton I, Jeyaseelan SM, Adaptation of a community-based type-2 diabetes mellitus remission intervention during COVID-19: empowering persons living with diabetes to take control. Implement Sci Commun. 2022 Jan 24;3:5. 35074020 10.1186/s43058-022-00255-9 PMC8785024

[20] Arena L, Soloe C, Schlueter D, Ferriola-Bruckenstein K, DeGroff A, Tangka F, Modifications in Primary Care Clinics to Continue Colorectal Cancer Screening Promotion During the COVID-19 Pandemic. J Community Health. 2023;48(1):113–26. 36308666 10.1007/s10900-022-01154-9 PMC9617236

[21] Martinez K, Lane E, Hernandez V, Lugo E, Muñoz FA, Sahms T, Optimizing ATTAIN Implementation in a Federally Qualified Health Center Guided by the FRAME-IS. Am Psychol. 2023;78(2):82–92. 37011161 10.1037/amp0001077 PMC10071441

[22] Baena A, Mesher D, Salgado Y, Martínez S, Villalba GR, Amarilla ML, Performance of visual inspection of the cervix with acetic acid (VIA) for triage of HPV screen-positive women: results from the ESTAMPA study. Int J Cancer. 2023 Apr 15;152(8):1581–92. 36451311 10.1002/ijc.34384 PMC10107773

[23] Randall TC, Sauvaget C, Muwonge R, Trimble EL, Jeronimo J. Worthy of further consideration: An updated meta-analysis to address the feasibility, acceptability, safety and efficacy of thermal ablation in the treatment of cervical cancer precursor lesions. Prev Med. 2019 Jan;118:81–91. 30342109 10.1016/j.ypmed.2018.10.006

[24] Ur Rashid MH, Ahmed MM, Chowdhury S, Ahmed S. Effectiveness of visual inspection with acetic acid as a test for cervical cancer screening. International Journal of Noncommunicable Diseases. 2017 Mar;2(1):3.

[25] Adefuye PO, Dada OA, Adefuye BO, Shorunmu TO, Akinyemi BO, Idowu-Ajiboye BO. Feasibility, acceptability, and effectiveness of visual inspection of the cervix with acetic acid and cryotherapy for dysplasia in Nigeria. International Journal of Gynecology & Obstetrics. 2015;129(1):62–6. 25593107 10.1016/j.ijgo.2014.10.032

[26] Shin MB, Oluoch LM, Barnabas RV, Baynes C, Fridah H, Heitner J, Implementation and scale-up of a single-visit, screen-and-treat approach with thermal ablation for sustainable cervical cancer prevention services: a protocol for a stepped-wedge cluster randomized trial in Kenya. Implementation Science. 2023 Jun 26;18(1):26. 37365575 10.1186/s13012-023-01282-3 PMC10294443

[27] CATEGORIZATION OF HEALTH INSTITUTIONS.

[28] Zeng W, Musiega A, Oyasi J, Di Giorgio L, Chuma J, Lu R, Understanding the performance of county health service delivery in Kenya: a mixed-method analysis. Health Policy Plan. 2022 Feb 8;37(2):189–99. 34718555 10.1093/heapol/czab129 PMC7613432

[29] East LA, Arudo J, Loefler M, Evans CM. Exploring the potential for advanced nursing practice role development in Kenya: a qualitative study. BMC Nurs. 2014 Nov 14;13:33. 25411562 10.1186/s12912-014-0033-y PMC4236483

[30] Miller CJ, Barnett ML, Baumann AA, Gutner CA, Wiltsey-Stirman S. The FRAME-IS: a framework for documenting modifications to implementation strategies in healthcare. Implement Sci. 2021 Apr 7;16(1):36. 33827716 10.1186/s13012-021-01105-3 PMC8024675

[31] Anticipating Changes That May Affect Intervention Fidelity [Internet]. Rethinking Clinical Trials. [cited 2025 Feb 19]. Available from: https://rethinkingclinicaltrials.org/chapters/conduct/monitoring-intervention-fidelity-and-adaptations/anticipating-changes-that-may-affect-intervention-fidelity/

[32] Adedimeji A, Ajeh R, Pierz A, Nkeng R, Ndenkeh JJr, Fuhngwa N, Challenges and opportunities associated with cervical cancer screening programs in a low income, high HIV prevalence context. BMC Womens Health. 2021 Feb 18;21:74. 33602194 10.1186/s12905-021-01211-w PMC7890622

[33] Holme F, Kapambwe S, Nessa A, Basu P, Murillo R, Jeronimo J. Scaling up proven innovative cervical cancer screening strategies: Challenges and opportunities in implementation at the population level in low- and lower-middle-income countries. Int J Gynaecol Obstet. 2017 Jul;138 Suppl 1:63–8. 28691331 10.1002/ijgo.12185

[34] Dsouza JP, Van den Broucke S, Pattanshetty S, Dhoore W. Cervical cancer screening status and implementation challenges: Report from selected states of India. Int J Health Plann Manage. 2022 Mar;37(2):824–38. 34716616 10.1002/hpm.3353

[35] Valenta S, Ribaut J, Leppla L, Mielke J, Teynor A, Koehly K, Context-specific adaptation of an eHealth-facilitated, integrated care model and tailoring its implementation strategies—A mixed-methods study as a part of the SMILe implementation science project. Front Health Serv. 2023 Feb 17;2:977564. 36925799 10.3389/frhs.2022.977564 PMC10012712

[36] Metz A, Kainz K, Boaz A. Intervening for sustainable change: Tailoring strategies to align with values and principles of communities. Front Health Serv. 2023 Jan 18;2:959386. 36925846 10.3389/frhs.2022.959386 PMC10012724

[37] Geng EH, Mody A, Powell BJ. On-the-Go Adaptation of Implementation Approaches and Strategies in Health: Emerging Perspectives and Research Opportunities. Annu Rev Public Health. 2023 Apr 3;44:21–36. 37010927 10.1146/annurev-publhealth-051920-124515

[38] Stirman SW, Baumann AA, Miller CJ. The FRAME: an expanded framework for reporting adaptations and modifications to evidence-based interventions. Implementation Science : IS [Internet]. 2019 [cited 2024 Jan 25];14. Available from: https://www.ncbi.nlm.nih.gov/pmc/articles/PMC6554895/10.1186/s13012-019-0898-y PMC6554895 31171014

[39] Gagnon J, Breton M, Gaboury I. Decision-maker roles in healthcare quality improvement projects: a scoping review. BMJ Open Qual. 2024 Jan 5;13(1):e002522. 10.1136/bmjoq-2023-002522 PMC10773379 38176953

[40] Pannick S, Sevdalis N, Athanasiou T. Beyond clinical engagement: a pragmatic model for quality improvement interventions, aligning clinical and managerial priorities. BMJ Qual Saf. 2016 Sep;25(9):716–25. 10.1136/bmjqs-2015-004453 PMC5013121 26647411

[41] Lyng HB, Macrae C, Guise V, Haraldseid-Driftland C, Fagerdal B, Schibevaag L, Balancing adaptation and innovation for resilience in healthcare – a metasynthesis of narratives. BMC Health Serv Res. 2021 Jul 31;21:759. 34332581 10.1186/s12913-021-06592-0 PMC8325788

[42] Proctor EK, Powell BJ, McMillen JC. Implementation strategies: recommendations for specifying and reporting. Implementation Science. 2013 Dec 1;8(1):139. 24289295 10.1186/1748-5908-8-139 PMC3882890

[43] Chambers DA. Advancing adaptation of evidence-based interventions through implementation science: progress and opportunities. Front Health Serv. 2023 Jun 5;3:1204138. 37342795 10.3389/frhs.2023.1204138 PMC10277471

